# Diagnostic accuracy, reliability, and construct validity of the German quick mild cognitive impairment screen

**DOI:** 10.1186/s12877-024-05219-3

**Published:** 2024-07-18

**Authors:** Patrick Manser, Eling D. de Bruin

**Affiliations:** 1 Motor Control and Learning Group - Institute of Human Movement Sciences and Sport, Department of Health Sciences and Technology, ETH Zurich, Zurich, Switzerland; 2 Department of Health, OST - Eastern Swiss University of Applied Sciences, St. Gallen, Switzerland; 3 Division of Physiotherapy, Department of Neurobiology, Care Sciences and Society, Karolinska Institute, Stockholm, Sweden

**Keywords:** Area under curve, Cognitive dysfunction, Dementia, Mild cognitive impairment, Neurocognitive disorders, ROC curve, Screening, Secondary prevention, Sensitivity and specificity

## Abstract

**Background:**

Early detection of cognitive impairment is among the top research priorities aimed at reducing the global burden of dementia. Currently used screening tools have high sensitivity but lack specificity at their original cut-off, while decreasing the cut-off was repeatedly shown to improve specificity, but at the cost of lower sensitivity. In 2012, a new screening tool was introduced that aims to overcome these limitations – the Quick mild cognitive impairment screen (Qmci). The original English Qmci has been rigorously validated and demonstrated high diagnostic accuracy with both good sensitivity and specificity. We aimed to determine the optimal cut-off value for the German Qmci, and evaluate its diagnostic accuracy, reliability (internal consistency) and construct validity.

**Methods:**

We retrospectively analyzed data from healthy older adults (HOA; *n* = 43) and individuals who have a clinical diagnosis of ‘mild neurocognitive disorder’ (mNCD; *n* = 37) with a biomarker supported characterization of the etiology of mNCD of three studies of the ‘Brain-IT’ project. Using Youden’s Index, we calculated the optimal cut-off score to distinguish between HOA and mNCD. Receiver operating characteristic (ROC) curve analysis was performed to evaluate diagnostic accuracy based on the area under the curve (AUC). Sensitivity, specificity, positive predictive value (PPV), and negative predictive value (NPV) were calculated. Reliability (internal consistency) was analyzed by calculating Cronbach’s α. Construct validity was assessed by analyzing convergent validity between Qmci-G subdomain scores and reference assessments measuring the same neurocognitive domain.

**Results:**

The optimal cut-off score for the Qmci-G was ≤ 67 (AUC = 0.96). This provided a sensitivity of 91.9% and a specificity of 90.7%. The PPV and NPV were 89.5% and 92.9%, respectively. Cronbach’s α of the Qmci-G was 0.71 (CI_95%_ [0.65 to 0.78]). The Qmci-G demonstrated good construct validity for subtests measuring learning and memory. Subtests that measure executive functioning and/or visuo-spatial skills showed mixed findings and/or did not correlate as strongly as expected with reference assessments.

**Conclusion:**

Our findings corroborate the existing evidence of the Qmci’s good diagnostic accuracy, reliability, and construct validity. Additionally, the Qmci shows potential in resolving the limitations of commonly used screening tools, such as the Montreal Cognitive Assessment. To verify these findings for the Qmci-G, testing in clinical environments and/or primary health care and direct comparisons with standard screening tools utilized in these settings are warranted.

**Supplementary Information:**

The online version contains supplementary material available at 10.1186/s12877-024-05219-3.

## Introduction

### Background

Three of the top ten current research priorities aimed at reducing the global burden of dementia are centered around the prevention, early identification, and mitigation of dementia risk factors [1]. In this context, it is imperative that significant emphasis is placed on improving the timely and accurate detection and diagnosis of mild and major neurocognitive disorder (mNCD and MNCD; formerly referred to as ‘mild cognitive impairment’ and ‘dementia’ [2–6]) to facilitate early interventions as part of the secondary prevention of mNCD [1].

Neurocognitive disorders are currently largely underdiagnosed, with a global pooled prevalence of undetected MNCDs of 61.7% in middle- and high-income countries. There are various possible explanations for this phenomenon. For instance, primary care physicians and health professionals may still consider cognitive difficulties a common trait of normal aging rather than a disability that necessitates specialized attention and support. As a result, they may hesitate to refer these patients to memory clinics for a clinical diagnosis [7]. The widespread and consistent use of a validated and recognized screening tool would undoubtedly improve the ability to detect individuals with suspected NCDs who should be referred for a clinical diagnosis [7], which is consistent with the majority of currently available clinical practice guidelines for the diagnosis and treatment of mNCD [8]. However, recommendations by the United States Preventive Services Task Force in 2020 and the Canadian Task Force on Preventive Health in 2016 do not suggest screening for cognitive impairment or dementia in asymptomatic older adults due to the lack of evidence demonstrating its advantages as well as potentially high rate of false-positive screens [9, 10].

The most frequent screening tools for individuals with suspected NCDs utilized in clinical practice and research [8, 11–13] include the Mini-Mental State Examination (MMSE) [14] and the Montreal Cognitive Assessment (MoCA) [15]. The MoCA was found the most common and preferable tool for screening of mNCD [13] and is superior to the MMSE in the detection of mNCD [16]. However, while the initially proposed cut-off (< 26 points) [15] has shown good sensitivity for discriminating mNCD from healthy older adults (HOA) [15, 17] with a pooled sensitivity of 93.7% [18], this cut-off was repeatedly found to have low specificity [17] (pooled specificity = 58.8%) [18]. Similar findings have been obtained for the original cut-off for the German MoCA, with a sensitivity and specificity of 86% and 63%, respectively [19]. Decreasing the cut-off was repeatedly shown to improve specificity, but at the cost of lower sensitivity. Therefore, it was recommended to adjust the utilized cut-off scores based on the preferred prioritization of sensitivity or specificity [18], which has been thoroughly investigated in the German MoCA [19]. Thomann et al. (2020) concluded that *“using two separate cut-offs for the MoCA combined with scores in an indecisive area enhances the accuracy of cognitive screening“* [19]. Alternatively, more robust and accurate screening tools should be developed [18].

In 2012, a new screening tool was introduced that aims to overcome these limitations – the Quick mild cognitive impairment screen (Qmci) [20–22]. In comparison to the MMSE and MoCA, the Qmci has a more detailed scoring system and includes a logical memory task, which allows it to detect subtle cognitive changes and avoid ceiling effect [23]. The original English Qmci was shown to accurately discriminate between individuals with normal cognitive functioning (*n* = 623), mNCD (*n* = 147), and MNCD (*n* = 165) [24]. In addition, the Qmci has undergone successful validation in multiple languages, including Chinese [25], Dutch [26], Greek [27, 28], Japanese [29], Persian [30], Taiwanese [31], and Turkish [32]. However, the optimal cut-off for discriminating between mNCD and HOA as well as the diagnostic accuracy of the German version of the Qmci (Qmci-G) have not yet been scientifically determined and validated. These investigations are required for the Qmci-G to be used in German-speaking countries.

### Objectives

The primary objective of this study was to determine the optimal cut-off value for the Qmci-G and to evaluate its diagnostic accuracy. As secondary objectives, we assessed the reliability (internal consistency) of the Qmci-G and explored the construct validity of the Qmci-G in older adults who have mNCD.

## Methods

### Study Design and participants

This study retrospectively analyzed data of three studies of the ‘Brain-IT’ project, namely baseline assessments of a cross-sectional study which included assessments of the Qmci-G in HOA [33] and two intervention studies that assessed the feasibility [34] and effectiveness ([35, 36]) of a novel technology-supported training concept for the secondary prevention of mNCD. The study was reported according to the Standards for Reporting of Diagnostic Accuracy Studies guidelines and elaboration paper [37, 38] (checklist see supplementary file 1).

In the cross-sectional study, HOA (healthy based on self-report and ≥ 60 years) were recruited between January 2021 and June 2021 in collaboration with healthcare institutions in the larger area of Zurich by handing out leaflets to interested persons. In the two intervention studies, older adults who have mNCD were recruited between July 2021 and October 2023 in collaboration with (memory) clinics in the larger area of Zurich and St. Gallen. All suitable patients were identified through medical records and patient registries at these (memory) clinics, or through recent clinical diagnostics. For this study, we only analyzed data of all participants who have a biomarker supported characterization of the etiology of mNCD in addition to the clinical diagnosis of ‘mild neurocognitive disorder’ according to International Classification of Diseases 11th Revision (ICD-XI) [40] or the latest Diagnostic and Statistical Manual of Mental Disorders 5th Edition (DSM-5^®^) [7]. Besides these inclusion criteria for the characterization of the population (HOA and mNCD), the same eligibility criteria were used in all three studies (for the full list of eligibility criteria, refer to Table 1).

**Table 1:** Description of all eligibility criteria

Inclusion criteria:	Exclusion criteria:
Participants fulfilling all the following inclusion criteria were eligible:	The presence of any of the following criteria led to exclusion:
• (1 = mNCD) clinical diagnosis of ‘mild neurocognitive disorder’ according to International Classification of Diseases 11th Revision (ICD-XI) [6] or the latest Diagnostic and Statistical Manual of Mental Disorders 5th Edition (DSM-5^®^) [5]) AND biomarker supported characterization of the etiology of mNCD OR (2 = HOA) healthy (based on self-report) older adults (≥ 60 years) • German speaking • able to stand for at least 10 min without assistance	• mobility impairments (i.e., gait, balance) that prevent experiment participation • presence of additional, clinically relevant (i.e., acute and/or symptomatic) neurological disorders (i.e., epilepsy, stroke, multiple sclerosis, Parkinson’s disease, brain tumors, or traumatic disorders of the nervous system) • presence of any other unstable or uncontrolled diseases (e.g., uncontrolled high blood pressure, progressing or terminal cancer)

Abbreviations: HOA, healthy older adults; mNCD, mild neurocognitive disorder

The first author (PM) was responsible for the design, implementation, conduct, and analysis of all three of these studies under the supervision of EdB. He trained each involved study investigator for all study procedures according to Guidelines of Good Clinical Practice and in line with detailed working instructions and was in charge of methodological standards and quality of data collection under the supervision of EdB. The same working instructions were followed in all three studies. These detailed working instructions standardized all measurement procedures and instructions of participants to minimize bias during assessment of all outcome measures.

### Outcomes

#### Primary outcome: Qmci

As primary outcome, data of the Qmci-G [20, 21] was used. The Qmci consists of six subtests: orientation (10 points), registration (5 points), clock drawing (15 points), delayed recall (20 points), verbal fluency (20 points), and logical memory (30 points) and is scored out of a maximum of 100 points [21, 22]. It was administered and evaluated according to published guidelines [21]. According to these guidelines, administration and scoring of the Qmci should not exceed 5 min [21].

#### Secondary outcomes

As secondary outcomes, data of assessments of the neurocognitive domains of learning and memory, executive functions, and visuo-spatial skills was used. For learning and memory, data of the German version of the subtest ‘logical memory’ of the Wechsler Memory Scale – fourth edition (WMS-IV-LM) [39, 40] was used. For executive functions, we considered data for working memory (i.e. using a computerized version of the Digit Span Backward test (Psychology Experiment Building Language (PEBL) - Digit Span Backward (PEBL-DSB)) [41–43], cognitive flexibility (i.e. using a computerized version of the Trail Making Test – Part B (PEBL-TMT-B) [41, 43]), and planning abilities (i.e. using the HOTAP picture-sorting test part A (HOTAP-A) [44]). For visuo-spatial skills, we considered data from a computerized Mental Rotation Task (PEBL-MRT) [41, 43, 45] that is based on the classic mental rotation task by Shepard and Metzler [46]. All assessments were administered and evaluated in accordance with published guidelines or detailed working instructions. For further information on these assessments, please refer to the study protocol of our RCT [36].

#### Other outcomes

Baseline factors were collected through demographic data including age, sex, height, weight, body mass index (BMI), years of education, and (for participants who have mNCD) classification of etiology of mNCD (biomarker supported).

### Statistics

Statistical analysis was done after data collection was completed using R (4.3.1 GUI 1.79 Big Sur Intel build) in line with RStudio (Version 2023.06.2 + 561). Data was reported as means ± standard deviations for data fulfilling all the assumptions that would subsequently justify parametric statistical analyses. In case these assumptions were not met, medians (interquartile ranges) were reported. First, descriptive statistics were computed for all outcome variables [47–49]. The normality of the data was checked using the Shapiro-Wilk test. For all demographic variables, between-group differences (i.e., HOA and older adults who have mNCD) were tested using an independent t-test or Mann–Whitney U-test in case the data were not normally distributed. Between-group differences in categorical variables were tested using Fisher’s exact test. To discover whether the between-group differences were substantive, Pearson’s r effect sizes were calculated [49, 50] and interpreted to be small (0.1 ≤ *r* < 0.3), medium (0.3 ≤ *r* < 0.5) or large (*r* > 0.5) [51]. The level of significance was set at *p* ≤ 0.05 (one-sided).

#### Optimal cut-off value and diagnostic accuracy of the Qmci-G

The optimal cut-off score for discriminating between HOA and older adults who have mNCD was calculated using Youden’s Index [52] in the OptimalCutpoints package [53]. Receiver operating characteristic (ROC) curve analysis was done using the pROC package [54] and used to assess diagnostic accuracy based on the area under the curve (AUC). The resulting AUC value was interpreted to represent poor (0.60 ≤ |AUC| < 0.70), fair (0.70 ≤ |AUC| < 0.80), good (0.80 ≤ |AUC| < 0.9), and excellent (|AUC| ≥ 0.90) discriminatory ability [55, 56]. Sensitivity, specificity, positive predictive value (PPV), and negative predictive value (NPV) were calculated for the optimal cut-off score.

#### Reliability (internal consistency) of the Qmci-G

Cronbach’s α was calculated to investigate the internal consistency of the Qmci-G [49]. The degree of consistency was interpreted according to the categorization for Cronbach’s α defined in [57]. Cronbach’s α ≥ 0.70 was set as the criterion for “adequate” internal consistency [57].

#### Construct validity of the Qmci-G in older adults who have mNCD

The Qmci-G subtest ‘orientation’ is mainly intended to distinguish between individuals who have mNCD and MNCD and therefore has limited discriminatory power between HOA and individuals who have mNCD due to ceiling effects [58, 59]. Therefore, assessment of construct validity focused on the remaining subtests of the Qmci in this study.

Construct validity of the Qmci-G was assessed by analyzing convergent validity between the Qmci-G subdomain scores and reference assessments measuring the same neurocognitive domain according to Sachdev et al. 2014 [3]. It was hypothesized that there is a significant strong positive correlation between: (alternative hypothesis number 1 (H_A,1_):) Qmci-G subtest ‘registration’ and reference assessments for auditory learning and memory; (H_A,2_:) Qmci-G subtest ‘clock drawing’ and reference assessments for executive functions/visuo-spatial skills; (H_A,3_:) Qmci-G subtest ‘recall’ and reference assessments for auditory learning and memory; (H_A,4_:) Qmci-G subtest ‘logical memory’ and reference assessments for auditory learning and memory; (H_A,5_:) Qmci-G subtest ‘verbal fluency’ and reference assessments for executive functions.

One-sided bivariate correlation analyses were performed for the neurocognitive domains of (1) learning and memory (i.e., between Qmci-G subscores ‘registration’, ‘delayed recall’ as well as ‘logical memory’ and WMS-IV-LM 1 and 2 [39, 40] for auditory learning and memory; (2) executive functions (i.e., Qmci-G subscores ‘verbal fluency’ and PEBL-DSB [41–43], PEBL-TMT-B [41, 43]), as well as HOTAP-A [44]; and (3) and combined executive functions/visuo-spatial skills (i.e., Qmci-G subscore ‘clock drawing’ and PEBL-DSB [41–43], PEBL-TMT-B [41, 43], as well as PEBL-MRT [41, 43, 45, 46]). Pearson’s correlation coefficients (r) were computed for datasets adhering to assumptions for parametric analyses and Spearman’s rank correlation coefficients (r_s_) for datasets violating assumptions for parametric analyses. 95% confidence intervals (CI_95%_) were calculated using the R-package ‘ci_cor’. For spearman correlation coefficients, we used bootstrap CI_95%_ with the bias-corrected and accelerated method, 999 bootstrap resamples and 1,000 seeds. The resultant correlation coefficients were interpreted as weak (0.1 ≤ |_r(s)_| < 0.3), moderate (0.3 ≤ |_r(s)_| < 0.5) or strong (|r_(s)_| ≥ 0.5) correlation [49, 51]. The alternative hypotheses (i.e., convergent validity between the Qmci-G subdomain scores and reference assessments measuring the same neurocognitive domain) were considered confirmed in case of: (a) a significant (*p* ≤ 0.05, one-tailed) positive correlation between the Qmci-G subdomain score and the corresponding reference assessment, and (b) a correlation coefficient of |r_(s)_| ≥ 0.4 [60].

### Sample size justification

In this study, we did not perform an a-priori sample size calculation as we analyzed existing datasets from studies conducted as part of the ‘Brain-IT’ project. This approach is supported by various factors and aligns with our research objectives.

Extensive data on the diagnostic accuracy of the original English Qmci is available. As summarized in the introduction, the original Qmci was shown to accurately discriminate between individuals with normal cognitive functioning, mNCD, and MNCD [24] with high diagnostic accuracy, sensitivity, and specificity. In addition, the Qmci has undergone successful validation in multiple languages, including Chinese [25], Dutch [26], Greek [27, 28], Japanese [29], Persian [30], Taiwanese [31], and Turkish [32]. These studies were adequately powered according to a priori sample size calculations. The robustness of our retrospective data analysis is validated due to the dataset’s similar sample size to most of these studies.

In this study, our aim was to corroborate and build upon these research findings referenced above. To this end, we primarily aimed to ensure that our study population was representative in terms of demographic characteristics as well as descriptive statistics of the Qmci to optimize generalizability of our findings and critically discuss whether this was successful in section ‘Discussion – Generalizability of the Findings’.

## Results

### Descriptive statistics of Study Population

The descriptive statistics of the study population are summarized in Table 2. There were no adverse events related to any of the study’s measurements.

**Table 2:** Demographic characteristics of the Study Population;

	Group 1: HOA (*n* = 43)	Group 2: mNCD (*n* = 37)	Between-Group Difference		
			test statistics^(1)^	*p*-value^(2)^	effect size [CI_95%_] ^(3)^
Age [years]	67.0 (7.0)	77.0 (10.0)	W = 423	< 0.001*	r_s_ = -0.402 [-0.571 to -0.120]
Sex [% females]	58.1	32.4	N/A	0.026*	OR = 2.9 [1.2 to 7.2]
Body mass index [kg·m^-2^]	23.7 ± 3.0	23.2 (3.9)	W = 829	0.750	r_s_ = -0.036 [-0.253 to 0.186]
Years of education [years]	15.9 ± 4.3	14.9 ± 4.0	t = 0.8	0.416	*r* = 0.107 [-0.142 to 0.342]
Etiology of mNCD:					
mNCD due to Alzheimer’s Disease	N/A	*n* = 25 (67.6%)	N/A	N/A	N/A
mild frontotemporal NCD	N/A	*n* = 3 (8.1%)	N/A	N/A	N/A
mNCD with Lewy Bodies	N/A	*n* = 1 (2.7%)	N/A	N/A	N/A
mild vascular NCD	N/A	*n* = 6 (16.2%)	N/A	N/A	N/A
unclear / not (yet) determined	N/A	*n* = 2 (5.4%)	N/A	N/A	N/A
Qmci-G total score	78.8 ± 8.6	54.5 ± 13.0	t = 10.3	< 0.001*	*r* = -0.799 [-0.872 to -0.691]
Qmci-G subscore ‘orientation’	10.0 (0.0)	10.0 (2.0)	W = 1,120	< 0.001*	r_s_ = -0.489 [-0.640 to -0.302]
Qmci-G subscore ‘registration’	5.0 (0.5)	4.0 (2.0)	W = 1,233	< 0.001*	r_s_ = -0.517 [-0.662 to -0.336]
Qmci-G subscore ‘clock drawing’	15.0 (0.0)	13.0 (2.0)	W = 1,400	< 0.001*	r_s_ = -0.690 [-0.790 to -0.555]
Qmci-G subscore ‘recall’	20.0 (4.0)	12.0 (8.0)	W = 1,297	< 0.001*	r_s_ = -0.562 [-0.696 to -0.391]
Qmci-G subscore ‘fluency’	12.0 ± 2.4	7.8 ± 2.3	t = 8.0	< 0.001*	*r* = -0.673 [-0.776 to -0.534]
Qmci-G subscore ‘logical memory’	20.4 ± 5.0	9.7 ± 6.2	t = 8.4	< 0.001*	*r* = -0.710 [-0.807 to -0.577]

Data is reported as means ± standard deviations for data fulfilling all the assumptions to justify parametric statistical analyses. In case these assumptions were not met, medians (interquartile ranges) are reported

^(1)^ t-statistics for the between-group differences tested with an independent t-test or Mann-Whitney U test in case the data are not normally distributed;

^(2)^*p*-values for the between-group differences tested with an independent t-test or Mann-Whitney U test in case the data are not normally distributed, or Fisher’s exact test for categorical variables

^(3)^ effect size estimates for the between-group differences tested with an independent t-test (effect size Pearson r) or Mann-Whitney U test (effect size Spearman rho (r_s_)) in case the data are not normally distributed, or Fisher’s exact test for categorical variables (odds ratio)

* = significant at *p* < 0.05.

Abbreviations: HOA, healthy older adults; mNCD, mild neurocognitive disorder; Qmci-G, German version of the Quick mild cognitive impairment screen

### Optimal cut-off value and diagnostic accuracy of the Qmci-G

**Fig. 1:**
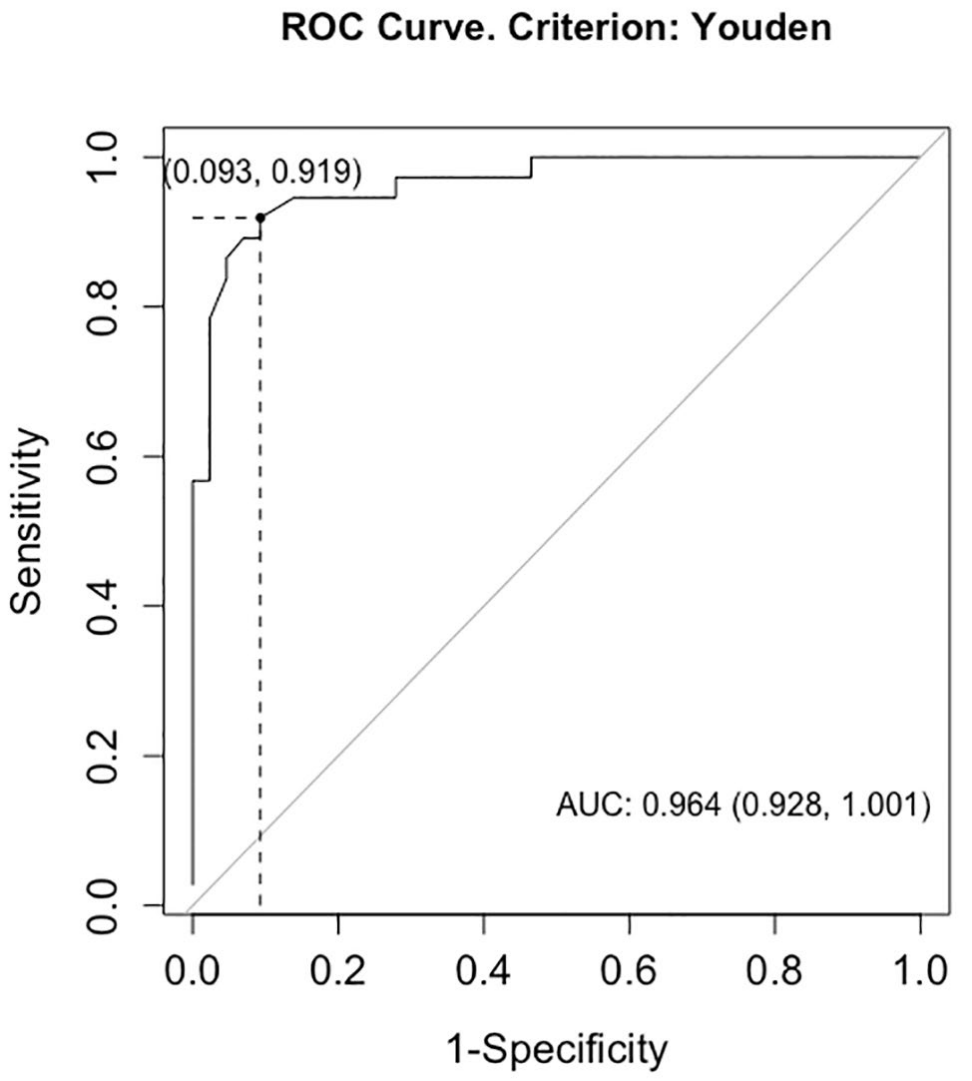
Receiver operating characteristic (ROC) curve with the optimal cut-off score for discriminating between healthy older adults and older adults who have mNCD calculated using Youden’s Index. Abbreviations: AUC, area under curve; ROC, receiver operating characteristic

Using Youden’s Index, the optimal cut-off score for the Qmci-G to discriminate between HOA and older adults who have mNCD was ≤ 67 (AUC = 0.96; 95% confidence interval (CI_95%_): 0.93, 1.00). This provided a sensitivity of 91.9% and a specificity of 90.7% (see Fig. 1). The PPV and NPV were 89.5% and 92.9%, respectively.

### Reliability (internal consistency) of the Qmci-G

Cronbach’s α of the Qmci-G was 0.71 (CI_95%_ [0.65 to 0.78]).

### Construct validity of the Qmci-G in older adults who have mNCD

The r_(s)_ and *p*-values for the correlation between the Qmci-G subtest scores and the scores of the corresponding reference assessments for each hypothesis are summarized in Table 3. The Qmci-G subtests assessing learning and memory showed a significant and strong correlation with the reference assessments. Subtests that measure executive functioning and/or visuo-spatial skills showed mixed findings and/or did not correlate as strong as expected with reference assessments.

**Table 3:** Bivariate correlation analyses between the German version of the quick mild cognitive impairment screen (Qmci-G) subtest scores and the scores of the corresponding reference assessments

Construct	Statistics	
	*p*-value^(1)^	*r*_(s)_ [CI_95%_]^(1)^
**Learning and Memory:**		
Qmci-G subscore ‘registration’ - WMS-IV-LM 1 (score)	0.013*	0.366 [-0.007 to 0.612]
Qmci-G subscore ‘registration’- WMS-IV-LM 2 (score)	< 0.001*	0.508 [0.207 to 0.724
Qmci-G subscore ‘delayed recall’ - WMS-IV-LM 1 (score)	< 0.001*	0.516 [0.183 to 0.742]
Qmci-G subscore ‘delayed recall’ - WMS-IV-LM 2 (score)	< 0.001*	0.604 [0.284 to 0.802]
Qmci-G subscore ‘logical memory’ - WMS-IV-LM 1 (score)	< 0.001*	0.721 [0.517 to 0.847]
Qmci-G subscore ‘logical memory’ - WMS-IV-LM 2 (score)	0.005*	0.414 [0.104 to 0.651]
**Executive Functions:**		
Qmci-G subscores ‘verbal fluency’- PEBL-DSB (score)	0.038*	0.312 [-0.035 to 0.592]
Qmci-G subscores ‘verbal fluency’- PEBL-TMT-B (time)	0.125	0.200 [-0.143 to 0.500]
Qmci-G subscores ‘verbal fluency’- HOTAP-A (combi-score)	0.149	0.178 [-0.159 to 0.479]
**Executive Functions/Visuo-spatial Skills:**		
Qmci-G subscores ‘clock drawing’ - PEBL-DSB (score)	0.001*	0.504 [0.136 to 0.748]
Qmci-G subscores ‘clock drawing’ - PEBL-TMT-B (time)	0.004*	0.437 [0.098 to 0.688]
Qmci-G subscores ‘clock drawing’ - PEBL-MRT (points)	0.170*	0.178 [-0.171 to 0.492]
Qmci-G subscores ‘clock drawing’ - PEBL-MRT (reaction time)	0.051	0.300 -0.067 to 0.582]

^(1)^ Pearson’s correlation coefficients (r) for datasets adhering to assumptions for parametric analyses, Spearman’s rank correlation coefficients (r_s_) for datasets violating assumptions for parametric analyses, and *p*-values

* = significant at *p* < 0.05

Abbreviations: CI_95%_, 95% confidence interval; DSB, Digit Span Backward; HOTAP-A, HOTAP picture-sorting test part A; MRT, Mental Rotation Task; PEBL, Psychology Experiment Building Language; Qmci, Quick mild cognitive impairment screen; TMT-B, Trail Making Test – Part B; WMS-IV-LM, subtest ‘logical memory’ of the Wechsler Memory Scale – fourth edition

## Discussion

This study determined the optimal cut-off value for the Qmci-G, and evaluated its diagnostic accuracy, reliability (internal consistency) and construct validity. The key findings of this study are that the Qmci-G demonstrated (1) excellent discriminatory ability between HOA and older adults who have mNCD at its optimal cut-off score of ≤ 67 points; (2) adequate internal consistency; and (3) good construct validity for subtests measuring learning and memory. However, subtests that measure executive functioning and/or visuo-spatial skills showed mixed findings and/or did not correlate as strongly as expected with reference assessments.

### Diagnostic accuracy of the Qmci

The excellent discriminatory ability of the Qmci-G between HOA and mNCD found in this study is consistent with extensive data on good diagnostic accuracy of the Qmci [11, 24] and, therefore, corroborates the available evidence of the original English Qmci (AUC = 0.84, optimal cut-off (Youden Index) ≤ 67, Sensitivity = 77%, Specificity = 75%) [24]. In addition, our findings are consistent with pooled data of 2019 for the Qmci demonstrating an AUC of 0.84 [11], a sensitivity between 77% [11] to 82% [23], and a specificity of 79% [11] to 82% [23] at a given cut-off score (i.e., the recommended cut-off score or, in case several sensitivity/specificity pairs were presented in the original studies analyses in these systematic reviews and meta-analyses, the cut-off score that was described as optimal by the respective authors or produced the largest AUC). Finally, our findings are also consistent with more recent validation studies of the Qmci in other translations, including Greek (AUC = 0.79 [28] and AUC = 0.76 [27]), Japanese (Sensitivity = 94%, Specificity = 72%) [29], Persian (AUC = 0.80) [30], Taiwanese (AUC = 0.89) [31], and Turkish (AUC = 0.80) [32]. The considerably higher AUC as compared to previous publications may be attributed to several characteristics of our analysis pertaining to the recruitment and characteristics of the participant sample under investigation. These are discussed in greater detail in the sections “Generalizability of the Findings” and “Strengths and Limitations”.

More importantly, there is evidence from a systematic review [23] and a meta-analysis [11] demonstrating that the Qmci has comparable [11] to superior [23] accuracy, sensitivity, and specificity compared to the standardized MMSE and the MoCA in detecting cognitive impairment [11, 23]. This finding holds significance for research and clinical practice, because the MMSE and MoCA are the most widely used screening instruments for mNCD [8, 12] and are known to have high sensitivity but low specificity at its original cut-off [16–18], also in the German MoCA (specificity = 63% at original cut-off) [19], while decreasing the cut-off was repeatedly shown to improve specificity, but at the cost of lower sensitivity [18]. In contrast, our results indicate both high sensitivity and specificity. This may be explained because the Qmci was developed on basis of the AB Cognitive Screen 135 [58] by reweighting its scoring and introducing a logical memory task with the aim to increase sensitivity and particularly specificity to detect mNCD [61]. This appears successful, since the logical memory subtest exhibited the highest accuracy of all Qmci subtests in discriminating between HOA and older adults with mNCD, as evidenced by an AUC of 0.80 [59]. Moreover, pooled sensitivity and specificity estimates of the Qmci were shown to not significantly differ from comprehensive cognitive assessments [11], such as the Addenbrooke’s Cognitive Examination Revised (ACE-R) [62], the Consortium to Establish a Registry for Alzheimer’s Disease Battery (CERAD) total score [63]. However, the Qmci has a substantially shorter administration time. According to published guidelines, administration and scoring should not exceed 5 min [21], which aligns with the median administration times of 4.5 min [64] to 5 min [11] reported in the literature. In contrast, the MoCA has a median administration time of 9.5 min [64] to 12 min [11], the ACE-R takes 12 to 20 min [11, 62] and the CERAD takes 20 to 30 min [11, 65]. This substantially shorter administration time, coupled with comparable [11] or even marginally superior [23] diagnostic accuracy suggests that the Qmci has potential as a means of assessing patients who present with cognitive complaints in primary care [11] and thereby allow more widespread and consistent use of a validated screening tool. This could ultimately aid in the early detection of individuals with suspected mNCD, facilitating their referral for clinical diagnosis [7], and supporting the implementation of interventions as part of the secondary prevention of mNCD, all of which are currently among the top ten research priorities in reducing the global burden of dementia [1].

### Reliability (internal consistency) and construct validity of the Qmci

The internal consistency of the Qmci-G was found adequate (Cronbach’s α ≥ 0.70 [57]). This is consistent with previous research as evidenced by Cronbach’s α values of 0.71 [28], 0.81 [30], 0.85 [31], 0.95 [20] and indicates that the subtests of the Qmci consistently and reliably assess the same underlying construct or concept (global cognitive functioning). With regards to the construct validity of the Qmci, previous studies have only analyzed the convergent validity between the Qmci total score and reference assessments [21, 28, 29, 31, 32, 66, 67]. These studies have shown significant strong positive correlations with the MoCA [31, 32] and significant weak [66], moderate [29], or strong [28, 31] positive correlations with the MMSE. In addition, the Qmci also showed significant strong correlations with a detailed neuropsychological battery (the standardized Alzheimer’s Disease Assessment Scale - cognitive subscale (SADAS-cog) [68, 69]) as well as the Clinical Dementia Rating scale [70] and was shown to be responsive to change over time [67]. This supports the construct validity of the Qmci and indicates that it could be used as a substitute for more comprehensive neuropsychological assessments in clinical trials [67].

As we were limited by the data available in this retrospective data analysis, we were unable to confirm these findings with the Qmci-G, due to unavailability of data from reference assessments for global cognitive functioning. However, we demonstrated construct validity for the Qmci subtest measuring learning and memory, as evidenced by significant and mostly strong correlations with clinically validated reference assessments for learning and memory. This is an important finding, as 55% of the maximum total score is allocated to the Qmci subtests measuring learning and memory (i.e., ‘registration’ (maximum 5 points), ‘recall’ (maximum 20 points), and ‘logical memory’ (maximum 30 points) [20, 21]. It is well known that the first line of prediction of mNCD is assessment of the neurocognitive domain of learning and memory [71]. Therefore, one potential explanation for why the Qmci may be more specific than the MoCA is due to the MoCA’s lesser emphasis on learning and memory. This domain only accounts for 16.7% of the maximum total score for the MoCA [15]. In addition to learning and memory, the neurocognitive domain of executive functions as well as visuo-spatial skills also serve as an important indicator for individuals who have mNCD [71]. However, our results only partly confirmed construct validity for the Qmci subtests measuring these neurocognitive domains (i.e., ‘verbal fluency’ and ‘clock drawing’). This finding is surprising, as the clock drawing test is the third most frequently cited screening test after the MoCA and MMSE [12]. However, although it has shown good reliability, validity is only fair to good [12], which aligns with our findings. In addition, the subtest ‘clock drawing’ was found least accurate (AUC = 0.57) in discriminating HOA from mNCD [59], suggesting that there exists potential for enhancing Qmci’s diagnostic accuracy by substituting the corresponding subtests with alternatives that exhibit better construct validity and discriminatory power. Similarly, the ‘verbal fluency’ subtest of the Qmci did not meet our criteria for verifying construct validity to measure executive functions, although these have been demonstrated to have predictive value in detecting mNCD and differentiating it from HOA [71]. Nonetheless, this subtest has been shown to be the second most accurate in discriminating HOA from mNCD with an AUC of 0.77 [59].

Although our results suggest that the Qmci subscores measuring learning and memory could be analyzed separately, it must be emphasized that this was not originally intended. Rather, the total Qmci score, which has demonstrated construct validity (as described above), should primarily be analyzed [21]. Future research should directly compare the Qmci-G total score to reference assessments to verify construct validity of the Qmci-G. In addition, future research should explore whether the Qmci’s diagnostic accuracy can be further enhanced by substituting some of the subtests with an alternative that exhibits better construct validity and discriminatory power.

### Generalizability of the findings

Compared to the extensive validation studies of the original English Qmci [24], our study populations were comparable regarding the descriptive statistics on age (for both HOA and mNCD), sex distribution (only for HOA), and descriptive data on the Qmci total score and subscores (only for HOA). Our population of individuals who have mNCD had a slightly lower total score compared to the English Qmci, which is explained by a lower score in the subtest ‘logical memory’ whereas the descriptive data on all other subtests were similar [59]. Nonetheless, we found the same optimal cut-off score compared to the English Qmci [24], whereas a large variation of optimal cut-off scores has been observed in other languages of the Qmci (Chinese = 55.5 [25], Dutch = 51.5 [26], Greek ≤ 51 [28] or ≤ 71 [27, 28], Japanese ≤ 61 [29], Persian ≤ 53 [30], Taiwanese = 51.5 [31], and Turkish ≤ 53 [32]). These differences are likely related to differences in socio-demographic factors and/or the small sample sizes of these studies.

In this regard, our study population had substantially more years of education (in both groups) and men were overrepresented in the group of individuals who have mNCD. While a higher level of education is a well-known early-life protective factor against mNCD and might be linked to better cognitive performance and higher cognitive reserve [72, 73], there was no relevant between-group difference in years of education which aligns with the extensive validation studies of the original English Qmci [24]. Nonetheless, previous research has shown that the optimal cut-off values as well as sensitivity and specificity of the Qmci differ between groups of varying educational levels [24]. Additionally, overrepresentation of men may influence generalizability of our findings, because women have a higher prevalence of non-amnestic mNCD [74]. However, most of our study participants had mNCD due to Alzheimer’s disease and previous research has shown that there were no significant sex differences in the prevalence or incidence of mNCD when all subtypes were combined [74]. The reason for our well-educated and men-dominated study population of individuals with mNCD might be related to a selection bias in recruitment of study participants, because this study was conducted in a research environment and only participants that were referred to us by our clinical collaboration partners could be considered. This might limit the generalizability of our findings, especially in individuals with low levels of education and to some degree also in women.

On the other hand, the distribution of etiologies of mNCD was representative, as approximately 60–90% of individuals with mNCD have Alzheimer’s disease etiology, mild vascular neurocognitive disorder is the second most common etiology of mNCD, and only about 5% have mild frontotemporal neurocognitive disorder etiology [5].

To summarize, our findings are generalizable to moderately to highly educated populations with all etiologies of mNCD and across a wide age range. However, the generalizability of our findings may be limited in less educated individuals, women, and non-research settings. Therefore, it seems of crucial importance to verify these findings for the Qmci-G by testing it in clinical environments and/or primary health care in more representative populations of individuals who have mNCD.

### Implications for Research and Clinical Practice

The Qmci presents as an interesting avenue for improving early detection of individuals with suspected mNCD thanks to its shorter administration time compared to the most commonly used screening tools for suspected NCDs [8, 11–13], coupled with comparable [11] or even marginally superior [23] diagnostic accuracy. Additionally, the published guidelines for the administration and evaluation of the Qmci [21] are well-developed and allow for easy administration, scoring, and interpretation. This promotes its widespread use in research and clinical settings. However, prior to the widespread implementation of the Qmci-G in clinical practice in German-speaking countries, it is crucial to verify our findings on good diagnostic accuracy, reliability, and construct validity in clinical settings and/or primary healthcare. In this regard, it is recommended that the test-retest reliability of the Qmci-G be assessed to calculate the minimal detectable difference and ultimately determine the minimum clinically relevant change. In addition, direct comparisons with standard screening tools commonly used in these settings, such as the German version of the MoCA, are necessary to determine whether the Qmci-G outperforms these screening tools. The potential for further enhancing the Qmci’s diagnostic accuracy should also be explored by substituting some of the subtests with alternatives that exhibit better construct validity and discriminatory power. Finally, the feasibility, acceptability, and effectiveness of the implementation of the Qmci in standard clinical practice should be investigated. These investigations have the potential to facilitate early detection of individuals with suspected mNCD and their referral for clinical diagnosis [7], which supports the implementation of lifestyle changes and/or interventions to ameliorate secondary prevention of mNCD and ultimately reduce the global burden of dementia [1].

### Strengths and limitations

The major strength of this study is that we only included data of study participants who have a biomarker supported characterization of the etiology of mNCD in addition to the clinical diagnosis of mNCD according to the ICD-XI [40] or DSM-5^®^ [7]. In addition, we included data of individuals who have different etiologies of mNCD. Both strengths increase the generalizability of our findings.

The study also has some key limitations that are worth mentioning. Most importantly, we did not conduct an a-priori sample size calculation, and the sample size was comparatively small. Such limitations may affect the robustness and generalizability of our findings, considering that the study was not designed to ensure adequate statistical power; however, our primary objective was to corroborate and extend previous research, and we carefully discussed the generalizability of our findings to ensure robust conclusions. Furthermore, we conducted a retrospective analysis of data obtained from three different studies, which involved diverse outcome assessors and additional assessments beyond those analyzed in this study. This could possibly introduce some bias to our findings. Nevertheless, the Qmci-G was consistently administered as one of the first three assessments, thereby mitigating potential participant fatigue-related bias. All additional evaluations were routinely conducted in the same standardized order. Additionally, we utilized consistent eligibility criteria and strictly adhered to specific working instructions to standardize all measurement procedures and participant instructions, minimizing bias during outcome measure assessment. Therefore, these limitations were not anticipated to significantly affect the findings. However, the study’s design may have introduced selection bias in participant recruitment, particularly among those with mNCD, due to the heightened barriers to enrolling in a 12-week intervention study as opposed to a typical cross-sectional study utilized for evaluating the diagnostic accuracy of a screening tool. This may explain the limited generalizability of our findings in individuals with lower levels of education. Finally, the standard statistical significance threshold of *p* ≤ 0.05 was utilized. To ensure a careful interpretation of the findings, we based our interpretation on predefined criteria that included effect size estimates with CI_95%_ combined with the significance level. Additionally, we calculated p-values only for differences between sociodemographic factors and secondary outcomes (i.e., construct validity analysis). Therefore, this limitation did not affect our primary findings.

## Conclusion

Our findings corroborate the existing evidence of the Qmci’s good diagnostic accuracy, reliability, and construct validity. Additionally, the Qmci shows potential in resolving the limitations of commonly used screening tools, such as the MoCA. To verify these findings for the Qmci-G, testing in clinical environments and/or primary health care and direct comparisons with standard screening tools utilized in these settings are warranted.

### Electronic supplementary material

Below is the link to the electronic supplementary material.

Supplementary Material 1

## Data Availability

The datasets analyzed during the current study are available in the Zenodo repository, https://doi.org/10.5281/zenodo.10122140.

## Abbreviations

ACE-R: Addenbrooke’s Cognitive Examination Revised
AUC: Area under the curve
BMI: Body mass index
CI_95%_: 95% Confidence Interval
CERAD: Consortium to Establish a Registry for Alzheimer’s Disease Battery
DSM-5^®^): Diagnostic and Statistical Manual of Mental Disorders 5th Edition
HOA: healthy older adults
HOTAP-A: HOTAP picture-sorting test part A
ICD-XI: International Classification of Diseases 11th Revision
MMSE: Mini-Mental State Examination
mNCD: mild neurocognitive disorder
MNCD: major neurocognitive disorder
MoCA: Montreal Cognitive Assessment
NCD: neurocognitive disorder
NPV: negative predictive value
PEBL: Psychology Experiment Building Language
PEBL-DSB: computerized version of the Digit Span Backward test
PEBL-MRT: computerized Mental Rotation Task
PEBL-TMT-B: computerized version of the Trail Making Test – Part B
PPV: positive predictive value
Qmci: Quick mild cognitive impairment screen
Qmci-G: German version of the Quick mild cognitive impairment screen
r: Pearson’s correlation coefficients
RCT: randomized controlled trial
ROC: Receiver operating characteristic
r_s_: Spearman’s rank correlation coefficients
SADAS-Cog: standardized Alzheimer’s Disease Assessment Scale - cognitive subscale
WMS-IV-LM: subtest ‘logical memory’ of the Wechsler Memory Scale – fourth edition
