# Supplementary material for: Diagnostic accuracy, reliability, and construct validity of the German quick mild cognitive impairment screen
Source: BMC Geriatr. 2024 Jul 18;24:613. doi: 10.1186/s12877-024-05219-3 (PMC11256646; doi:10.1186/s12877-024-05219-3)
Supplement: Supplementary file 1 — Supplementary Material 1 [file 12877_2024_5219_MOESM1_ESM.pdf]

ORIGINAL RESEARCH

Manser et al.

Supplementary Files to Publication:

Diagnostic Accuracy, Reliability, and Construct Validity of the German Quick Mild Cognitive Impairment Screen

Patrick Manser*[1] ORCID: 0000-0003-3300-6524

patrick.manser@hest.ethz.ch

Eling D. de Bruin[1 - 3] ORCID: 0000-0002-6542-7385

eling.debruin@hest.ethz.ch

*Corresponding Author

[1] Motor Control and Learning Group - Institute of Human Movement Sciences and Sport, Department of Health Sciences and Technology, ETH Zurich, Zurich, Switzerland;

[2] Department of Health, OST - Eastern Swiss University of Applied Sciences, St. Gallen, Switzerland;

[3] Division of Physiotherapy, Department of Neurobiology, Care Sciences and Society, Karolinska Institute, Stockholm, Sweden

Supplementary File 1 - Standards for Reporting of Diagnostic Accuracy Studies Checklist [1, 2]

Section & Topic	No	Item	Reported in section:
**TITLE OR ABSTRACT**			
	**1**	Identification as a study of diagnostic accuracy using at least one measure of accuracy (such as sensitivity, specificity, predictive values, or AUC)	'Abstract'
**ABSTRACT**			
	**2**	Structured summary of study design, methods, results, and conclusions (for specific guidance, see STARD for Abstracts)	'Abstract'
**INTRODUCTION**			
	**3**	Scientific and clinical background, including the intended use and clinical role of the index test	'1.1 Background'
	**4**	Study objectives and hypotheses	'1.1 Background' and '2.3.3 Construct Validity of the Qmci-G in Older Adults who have mNCD'
**METHODS**			
Study design	**5**	Whether data collection was planned before the index test and reference standard were performed (prospective study) or after (retrospective study)	'2.1 Study Design and Participants'
Participants	**6**	Eligibility criteria	'2.1 Study Design and Participants'
	**7**	On what basis potentially eligible participants were identified (such as symptoms, results from previous tests, inclusion in registry)	'2.1 Study Design and Participants'
	**8**	Where and when potentially eligible participants were identified (setting, location and dates)	'2.1 Study Design and Participants'
	**9**	Whether participants formed a consecutive, random or convenience series	'2.1 Study Design and Participants'
Test methods	**10a**	Index test, in sufficient detail to allow replication	'2.2.1 Primary Outcome: Qmci'
	**10b**	Reference standard, in sufficient detail to allow replication	N/A (no reference standard as explained in '4.5 Strengths and Limitations')
	**11**	Rationale for choosing the reference standard (if alternatives exist)	N/A (no reference standard as explained in '4.5 Strengths and Limitations')
	**12a**	Definition of and rationale for test positivity cut-offs or result categories of the index test, distinguishing pre-specified from exploratory	'2.3.1 Optimal Cut-off value and Diagnostic Accuracy of the Qmci-G'
	**12b**	Definition of and rationale for test positivity cut-offs or result categories of the reference standard, distinguishing pre-specified from exploratory	N/A (no reference standard as explained in '4.5 Strengths and Limitations')
	**13a**	Whether clinical information and reference standard results were available to the performers/readers of the index test	N/A (no reference standard as explained in '4.5 Strengths and Limitations')
	**13b**	Whether clinical information and index test results were available to the assessors of the reference standard	N/A (no reference standard as explained in '4.5 Strengths and Limitations')
Analysis	**14**	Methods for estimating or comparing measures of diagnostic accuracy	'2.3.1 Optimal Cut-off value and Diagnostic Accuracy of the Qmci-G'
	**15**	How indeterminate index test or reference standard results were handled	N/A (no indeterminate index test data and no reference standard test as explained in '4.5 Strengths and Limitations')
	**16**	How missing data on the index test and reference standard were handled	N/A (no missing data on index test and no reference standard test as explained in '4.5 Strengths and Limitations')

	**17**	Any analyses of variability in diagnostic accuracy, distinguishing pre-specified from exploratory	'2.3.1 Optimal Cut-off value and Diagnostic Accuracy of the Qmci-G'
	**18**	Intended sample size and how it was determined	'2.4 Sample Size Justification'
**RESULTS**			
Participants	**19**	Flow of participants, using a diagram	N/A (secondary data analysis as explained in '2.1 Study Design and Participants')
	**20**	Baseline demographic and clinical characteristics of participants	'3.1 Descriptive Statistics of Study Population'
	**21a**	Distribution of severity of disease in those with the target condition	N/A (all study participants were clinically diagnosed with 'mild neurocognitive disorder' as described in '2.1 Study Design and Participants')
	**21b**	Distribution of alternative diagnoses in those without the target condition	N/A (no data available)
	**22**	Time interval and any clinical interventions between index test and reference standard	N/A (no reference test as explained in '4.5 Strengths and Limitations')
Test results	**23**	Cross tabulation of the index test results (or their distribution) by the results of the reference standard	'3.1 Descriptive Statistics of Study Population' (no reference standard as explained in '4.5 Strengths and Limitations')
	**24**	Estimates of diagnostic accuracy and their precision (such as 95 % confidence intervals)	'3.2 Optimal Cut-off value and Diagnostic Accuracy of the Qmci-G'
	**25**	Any adverse events from performing the index test or the reference standard	N/A (no adverse events)
**DISCUSSION**			
	**26**	Study limitations, including sources of potential bias, statistical uncertainty, and generalisability	'4.3 Generalizability of the Findings' and '4.5 Strengths and Limitations'
	**27**	Implications for practice, including the intended use and clinical role of the index test	'4.4 Implications for Research and Clinical Practice'
**OTHER INFORMATION**			
	**28**	Registration number and name of registry	'2.1 Study Design and Participants'
	**29**	Where the full study protocol can be accessed	N/A (no study protocol because this was a secondary data analysis as explained in '2.1 Study Design and Participants')
	**30**	Sources of funding and other support; role of funders	'6.3.2 Funding'

1 References

1. Bossuyt PM, Reitsma JB, Bruns DE, Gatsonis CA, Glasziou PP, Irwig L, Lijmer JG, Moher D, Rennie D, de Vet HCW *et al*: **STARD 2015: an updated list of essential items for reporting diagnostic accuracy studies**. *BMJ : British Medical Journal* 2015, **351**:h5527.
2. Cohen JF, Korevaar DA, Altman DG, Bruns DE, Gatsonis CA, Hooft L, Irwig L, Levine D, Reitsma JB, Vet HCWd *et al*: **STARD 2015 guidelines for reporting diagnostic accuracy studies: explanation and elaboration**. *BMJ Open* 2016, **6**(11):e012799.
